# Monitoring in the Intensive Care

**DOI:** 10.1155/2012/473507

**Published:** 2012-08-27

**Authors:** Eric Kipnis, Davinder Ramsingh, Maneesh Bhargava, Erhan Dincer, Maxime Cannesson, Alain Broccard, Benoit Vallet, Karim Bendjelid, Ronan Thibault

**Affiliations:** ^1^Department of Anesthesiology and Critical Care, Lille University Teaching Hospital, Rue Michel Polonowski, 59037 Lille, France; ^2^Department of Anesthesiology and Perioperative Care, University of California Irvine, Irvine, CA 92697, USA; ^3^Division of Pulmonary and Critical Care Medicine, Department of Medicine, University of Minnesota, Minneapolis, MN 55455, USA; ^4^Geneva Medical School, 1211 Geneva 14, Switzerland; ^5^Centre de Recherche en Nutrition Humaine Auvergne, UMR 1019 Nutrition Humaine, INRA, Clermont Université, Service de Nutrition Clinique, CHU de Clermont-Ferrand, 63009 Clermont-Ferrand, France; ^6^Intensive Care Division, Geneva University Hospitals, 1211 Geneva 14, Switzerland

## Abstract

In critical care, the monitoring is essential to the daily care of ICU patients, as the optimization of patient's hemodynamic, ventilation, temperature, nutrition, and metabolism is the key to improve patients' survival. Indeed, the decisive endpoint is the supply of oxygen to tissues according to their metabolic needs in order to fuel mitochondrial respiration and, therefore, life. In this sense, both oxygenation and perfusion must be monitored in the implementation of any resuscitation strategy. The emerging concept has been the enhancement of macrocirculation through sequential optimization of heart function and then judging the adequacy of perfusion/oxygenation on specific parameters in a strategy which was aptly coined “goal directed therapy.” On the other hand, the maintenance of normal temperature is critical and should be regularly monitored. Regarding respiratory monitoring of ventilated ICU patients, it includes serial assessment of gas exchange, of respiratory system mechanics, and of patients' readiness for liberation from invasive positive pressure ventilation. Also, the monitoring of nutritional and metabolic care should allow controlling nutrients delivery, adequation between energy needs and delivery, and blood glucose. The present paper will describe the physiological basis, interpretation of, and clinical use of the major endpoints of perfusion/oxygenation adequacy and of temperature, respiratory, nutritional, and metabolic monitorings.

## 1. Central Hemodynamic Monitoring

### 1.1. Introduction

In critical care, the optimization of patient's hemodynamic and temperature is the key to improve patient morbidity and mortality. The goal of hemodynamic monitoring is to provide data that aids in the optimization of end organ tissue oxygenation and effectively combats global tissue hypoxia, shock, and multiorgan failure. Traditional, noninvasive methods of hemodynamic monitoring pertained solely to physical examination, and invasive methods included central venous and pulmonary artery catheterization mostly. These pressure-derived preload values have been used extensively in the management of fluid resuscitation and titration. However, numerous studies of various patient populations (sepsis, cardiovascular surgery, trauma, and other critical illnesses) have challenged the notion that these indicators accurately predict volume status [1–7]. These “static” pressure-derived values do not accurately identify a position on the Starling curve and, therefore, poorly predict whether volume will improve hemodynamics. In fact a recent meta analysis showed no positive association between PAC use for fluid management and survival [8].

Recently, however, technologic advancements in this area have introduced new methods of noninvasive and less invasive hemodynamic monitoring. Generally, this data provides insight into the fluid status of the patient by indicating where the patient is on the Frank-Starling curve (preload) and may also provide insight into cardiac output, myocardial contractility, systemic vascular resistance, and more novel parameters related to the pulmonary vascular system. This chapter seeks to provide an overview of these new technologies and its implication in the critical care setting. 

### 1.2. Macrocirculation Monitoring

Identification of patients who are on the steep part of frank starling curve and therefore are fluid responsive is a core principle of hemodynamic monitoring and aids in the determination of the extent that circulatory homeostasis can be maintained with fluids alone, versus the need for inotropes or vasopressors. Similarly the continuous assessment of cardiac output, myocardial contractility, and vascular tone is crucial to the diagnosis and management of critically ill patients, and this has long been solely provided by the PAC catheter. Recently, however there are new technologies that may provide this information in a less invasive or completely noninvasive manner.

#### 1.2.1. Pulse Contour Analysis

The concept of pulse contour analysis is a method of ascertaining the cardiac output from analyzing of the pulse pressure waveform. It is known that the pulse pressure is directly proportional to stroke volume and inversely related to vascular compliance. Also it is known that the pulse pressure waveform depicts the changes in stroke volume that occur with positive pressure ventilation. Specifically, during the inspiratory phase of positive pressure ventilation, intrathoracic pressure increases passively, increasing right atrial pressure and causing venous return to decrease, decreasing right ventricular output, and after two or three heart beats affecting left ventricular output. Monitoring this stroke volume variation has shown to accurately predict patients who are fluid responsive [9]. A large pulse pressure/stroke volume variation (10% to 15%) is indicative of hypervolemia and predictive of volume responsiveness.

There are several technologies that use pulse contour analysis; these include the FloTrac, PiCCO, and LiDCO plus systems. These systems differ in their modality to assess for vascular tone their requirements for invasive monitoring and need for external calibration for CO measurements. A short discussion of each of these devices is in the following.

#### 1.2.2. The Vigileo/FloTrac System

The FloTrac has a proprietary software algorithm that analyzes characteristics of the arterial pressure waveform and uses this analysis, along with patient-specific demographic information, to determine continuous CO, systemic vascular resistance, and the dynamic parameter of stroke volume variation. It carries the advantage of being able to be used for any arterial catheter in any arterial location. In addition, the device self-calibrates were based on patient demographics and waveform analysis. Differences in patient populations, study environments (intraoperative, postoperative, nonsurgical), FloTrac software versions, ventilatory settings, medical interventions, and reference standard(s) used (intermittent thermodilution CO, continuous thermodilution CO, esophageal Doppler, PiCCO), combined with the relatively small single center studies, are all central to this issue. Newer FloTrac software versions have improved the accuracy of the system's ability to determine CO. 

#### 1.2.3. LiDCO Plus System

This system also uses analysis of pulse contour from an arterial line to determine stroke volume and CO. However, the main difference is that this system uses a lithium-based dye-dilution technique to calibrate its pulse contour analysis algorithm, referred to as Pulse CO. After calibration, the LiDCO plus system can generate CO measurements using pulse contour analysis; however recalibration is recommended every 8 hours. 

#### 1.2.4. The PiCCO System

Like the LiDCO and FloTrac systems, this device provides CO through pulse contour analysis of the arterial waveform. It also requires an external calibration (cold saline) for this analysis. The PiCCO monitor provides several other measurements as well including global end-diastolic volume measurements of all four heart chambers as well as extravascular lung water measurements. One of the limitations of this technology is the requirement for proximal artery catheterization with a thermistor-tipped catheter [10]. As with the other pulse contour technologies previously described, periods of significant hemodynamic instability result in potentially intolerable inaccuracies in CO measurement requiring frequent recalibration [11]. Once again, small single center studies, different settings, and different standards of reference make generalizations difficult.

#### 1.2.5. Esophageal Doppler

The esophageal Doppler is a flexible probe that has a Doppler transducer (4 MHz continuous wave or 5 MHz pulsed wave, according to manufacturers) at the tip that is placed in the esophagus to obtain an aortic velocity signal in the descending aorta. The technology allows one to gain insight into preload by looking at the flow time of the velocity time integral (VTI) of the aortic flow (normal = 330–360 msec), with states of decreased preload shortening the flow time. Also it quantifies myocardial contractility by assessing the peak velocity of the aortic VTI signal (normal > 70 cm/sec). Finally, this technology derives vascular tone by analysis of the VTI waveform. A meta-analysis Dark and Singer demonstrated an 86% correlation between cardiac output as determined by esophageal Doppler and PAC [12]. Clinical studies comparing TED guided protocols to conventional approaches of volume replacement (guided by clinical assessment and/or central venous pressure) conclusively report beneficial effects in the Doppler-optimized groups, including a reduced risk of postoperative morbidity and a shorter length of hospital or ICU stay [13–20]. However, the resulting waveform is highly dependent on correct positioning and requires frequent adjustments in depth, orientation, and gain to optimize the signal [21]. Therefore, while esophageal Doppler has some utility in aiding in the assessment of the hemodynamic status of critically ill patients, this technology has been slow to be adopted. This is likely secondary to high amount continuous user involvement needed to produce accurate data. 

#### 1.2.6. Thoracic Electrical Bioimpedance

Using low voltage, electrical impedance (or resistance) across the chest is measured. The higher the fluid content, the lower the impedance since fluid conducts electricity. As the volume of blood in the thorax changes during the heart cycles through systole and diastole, these variations can be measured electrically [22]. Many of the problems associated with TEB have been overcome with newer generation devices. Recently, a number of investigators have reported a good correlation between TEB and thermodilution in patients following cardiac surgery using these improved devices [23–28]. There are limited data on the use of TEB in critically ill ICU patients; however, the improved TEB technology does hold promise in this group of patients.

#### 1.2.7. Echocardiography

Recent advances in point of care ultrasound devices have tremendously increased the utility of echocardiography in the critical care setting. The benefit of echocardiography lies in the fact that it allows the clinician to directly visualize the cardiac anatomy as well assess flow dynamics and thus rapidly assess structural abnormalities, contractility, and intravascular volume. While historically echocardiography has required extensive specialty training, recent literature supports the ability to train noncardiologist to perform and interpret a limited transthoracic echocardiography exam [29, 30]. Recently, guidelines have been published for POC cardiac ultrasound by noncardiologists for the intensive care setting [30]. Some of key points from these guidelines include (1) CVP estimate via inferior vena cava (IVC) diameter and its response to respirations, (2) estimation of preload via right and left ventricular end diastolic diameters, (3) assessment of RV/LV function via fractional area change and detection of regional wall motion abnormalities, (4) recognition of pericardial effusion and tamponade, and (5) global assessment of valvular function via color Doppler interrogation. 

In summary, no device stands out as being better than another and although not perfectly accurate, all the devices are able to detect alterations in cardiac output. Therefore, the true benefit lies with correct application of these devices by understanding the technology as well as the limitations for each device.

## 2. Peripheral Hemodynamic-Tissue Perfusion Monitoring

Shock is defined as “inadequate tissue oxygen for aerobic cellular respiration.” Therein lie the issues of shock management: the relationship between oxygen delivery and perfusion, the issues of mitochondrial dysfunction and lactate, and the issue of inadequate delivery to demand. Shock results from varying macrocirculatory and microcirculatory failure leading to hypoperfusion. Additionally, mitochondrial dysfunction may result in cellular oxygen misuse. Furthermore, stress and physiological compensation increase oxygen demand in situations of poor delivery. This oxygen delivery and demand inadequacy compound organ failure and can ultimately result in death despite optimal management. 

Shock management has included “restoring” or “maximizing” oxygen delivery and tissue oxygenation, albeit with varying results. A meta-analysis [31] showed that mortality decreased and oxygen delivery increased when management was guided by endpoints such as central venous pressure (CVP), mean arterial pressure (MAP), cardiac output (CO), cardiac index (CI), oxygen transport (TO_2_), and central or mixed venous oxygen saturation (ScvO_2_ or SvO_2_). 

Rather than a “holy grail” endpoint, the past decade has been marked by the early goal directed therapy (EGDT) approach of Rivers [32]. EGDT is based upon sequential endpoints: CVP >8 mmHg, subsequent norepinephrine management to MAP >65 mmHg, followed by a global endpoint, ScvO_2_, to assess oxygen delivery adequacy. A >5% drop in ScvO_2_ led to Hb level assessment/transfusion, CO assessment/inotropes, or intubation, ventilation, and sedation to decrease O_2_ demand. Interestingly, EGDT led to increased fluid loading, blood transfusion, and inotropes. Regardless of controversies [33], EGDT has been integrated into many studies, recommendations, and other settings such as high-risk surgery [34–36]. 

However, impaired oxygen extraction in sepsis and altered flow impede the use of ScvO_2_ to assess adequate tissue perfusion/oxygenation [37], and high ScvO_2_ can coexist with hypoperfusion [38]. Therefore, beyond restoring ScvO_2_ >70%, judging tissue perfusion may require other parameters such as lactate clearance or venoarterial PCO_2_ gradient and/or the visualization of microcirculation.

### 2.1. Microcirculation Monitoring

An important subject characterizing critically ill patients is that capillary circulation cannot be predicted by macrohemodynamic parameters. As depicted in situations like septic shock [39, 40] or heart failure [41], despite an optimal macroperfusion (blood pressure, cardiac output, etc.), microcirculatory perfusion could be inadequate [42] and capillary flow severely altered and responsible for a persistent tissue ischemia. Using Sidestream dark-field (SDF) imaging [43], microcirculatory flow can be visualised at the bedside, noninvasively, in different tissue regions (sublingual, rectal mucosa, etc.). Hence, microcirculatory assessment becomes a part of the global hemodynamic evaluation in critically ill patients, since patient standard of care could be influenced. However, it is important to highlight that microcirculatory monitoring with SDF could be difficult as it has its own limitations regarding measurement errors [44]. As example, different recordings of 20 seconds should be performed in different locations and microcirculatory quantification should be based on the average of multiple recordings, each being performed by two independent investigators. Indeed, sometimes the result presented (MFI, capillary density, etc.) must be taken with caution for the present semiquantitative technique. Optimistically, in the future, new technology and measurement method should be developed to allow rapid, accurate, and reproducible assessment of capillary perfusion at the bedside.

### 2.2. Gastric Tonometry and Sublingual Capnography

It is a known phenomenon that early on in hemodynamic stressed states there is a flow distribution away from the gastrointestinal tract, resulting in an increase in the PCO_2_ of the stomach wall. It is assumed that the increased gastric mucosal CO_2_ leading to gastric mucosal acidosis is a result of anaerobic metabolism consequent to splanchnic hypoperfusion. Previous studies indicate that gastric tonometry is a highly sensitive predictor of outcome in patients undergoing cardiac surgery [45], admitted to the ICU [46], in sepsis [47], or with acute circulatory failure [48]. However, the widespread application of gastric tonometry has proven to be practically difficult. While these studies support the importance of assessing gastrointestinal perfusion, there are several limitations to gastric tonometry that impede its clinical implementation. First gastric tonometry relies on the concept that intraluminal gut CO_2_ will be elevated when local perfusion is compromised secondary to resulting anaerobic cellular metabolism from reduced oxygen delivery. In addition, the concept has yielded a very poor specificity secondary to multiple confounders such as inappropriate measurement of stomach content PCO_2_, temperature (Haldane effect) buffering of gastric acid by duodenal/esophageal reflux, difference in arterial supply, and enteral feeding. 

Recently sublingual capnometry has been introduced as a method of resolving many of these difficulties associated with gastric tonometry. Sublingual capnometry is a technically simple, noninvasive, inexpensive technology that has been shown to provide insight into the adequacy of tissue perfusion during both hemorrhagic and septic shock [37, 49, 50]. Further studies with this technology, however, are needed that demonstrate the clinical utility of PsiCO_2_ monitoring.

### 2.3. Tissue Oximetry

The assessment of end organ oxygenation may be of value when caring for the critically ill patient. Previous studies have shown that impaired tissue oxygenation events are not easily detected by usual monitoring of heart rate, urine output, central venous pressure (CVP), cardiac output (CO), and blood pressure (BP) [51, 52] secondary to compensatory autonomic mechanisms, such as regional vasoconstriction. Based on this concept one may be able to detect these compensatory stress states by assessing the microcirculatory status, such as the noninvasive measurement of tissue oxygen saturation (StO_2_) when coupled with a functional hemodynamic monitoring test, such as the vascular occlusion test (VOT). Noninvasive measurement of StO_2_ using near infrared spectroscopy (NIRs) has been shown as a valid method to assess the microcirculation status, especially in septic and trauma patients [53]. The addition of dynamic ischemic challenge in which VOT is utilized has shown to improve the predictability of StO_2_ to identify tissue hypoperfusion [54].

Similarly, the ability to continuously assess oxygen delivery to organs supplied by the splanchnic circulation may be of critical importance since blood flow abnormalities to this region are associated with a range of morbidities, perhaps most notably multiple organ failure that can lead to death [51]. Markers such as mixed venous saturation (SvO_2_) and serum lactate levels are markers of global oxygen supply and demand and may be a poor reflection of splanchnic regional oxygen delivery and regional tissue viability [51, 55–57]. One can postulate that detection of decreased splanchnic circulation by monitoring oxygen delivery to an organ system supplied by the splanchnic circulation would allow treatment of the causative physiologic state before more systemic measures (SvO_2_, lactate, HR, UOP, BP, CVP) are affected. Preliminary data with an esophageal probe T-STAT 303 (Spectros Corporation, Portola Valley, CA, USA) utilizing visible light spectroscopy (VLS) has shown positive results with its ability to detect ischemia to the splanchnic bed [58, 59].

### 2.4. Mixed Venous or Central Venous Oxygen Saturation (SvO_2_/ScvO_2_)

#### 2.4.1. SvO_2_ and Oxygen Extraction

In normal SaO_2_ and Hb conditions, SvO_2_ should be >70%. During effort, oxygen uptake increases with transport. The oxygen transport (TO_2_) and uptake (VO_2_) relationship is defined by the extraction ratio of oxygen (ERO_2_):

(1) $$\begin{array}{c} \text{ER}{\text{O}}_{2}=\frac{{\text{VO}}_{2}}{{\text{TO}}_{2}}. \end{array}$$

Through transformations

(2) $$\begin{array}{c} {\text{ERO}}_{2}=\frac{\text{CO}\times \left({\text{CaO}}_{2}-{\text{CvO}}_{2}\right)}{{\text{TO}}_{2}},\\ {\text{ERO}}_{2}=\frac{\text{CO}\times \left({\text{CaO}}_{2}-{\text{CvO}}_{2}\right)}{\left(\text{CO}\;\times \;{\text{CaO}}_{2}\right)},\\ {\text{ERO}}_{2}=\frac{\left({\text{CaO}}_{2}-{\text{CvO}}_{2}\right)}{{\text{CaO}}_{2}},\\ {\text{ERO}}_{2}=1-\frac{{\text{CvO}}_{2}}{{\text{CaO}}_{2}},\\ {\text{ERO}}_{2}=1-\frac{{\text{SvO}}_{2}}{{\text{SaO}}_{2}},\\ \frac{{\text{ERO}}_{2}}{{\text{SaO}}_{2}}=\frac{1}{{\text{SaO}}_{2}}-{\text{SvO}}_{2}. \end{array}$$

The resulting equation is

(3) $$\begin{array}{c} {\text{SvO}}_{2}=\frac{1}{{\text{SaO}}_{2}}-\frac{{\text{ERO}}_{2}}{{\text{SaO}}_{2}}. \end{array}$$

When arterial oxygenation is achieved, SaO_2_ is 100%:

(4) $$\begin{array}{c} {\text{SvO}}_{2}=1-{\text{ERO}}_{2}. \end{array}$$

Thus, normal SvO_2_ values of 70–75% correspond to normal ERO_2_ of 25–30% delivered oxygen. Oxygen extraction depends on activity, tissue, and mitochondrial function. During effort, increased oxygen demand leads to increased extraction and decreased SvO_2_. While SvO_2_ normally drops to 60% through ERO_2_ increase to 40%, SvO_2_ may drop to 40% with ERO_2_ reaching up to a maximum of 60%. If this ERO_2_MAX is reached, any further demand leads to anaerobic lactate production. This maximal “critical ERO_2_” corresponds to a “critical SvO_2_” of 40% below which inadequate transport-to-demand, and therefore shock, is inevitable [60].

#### 2.4.2. Interpreting SvO_2_

SvO_2_ is the net result of pathophysiological processes and therapeutic compensations of VO_2_ and TO_2_ (Table 1). Before ascribing ScvO_2_ decrease to VO_2_ increase and decreasing it, all causes of TO_2_ increase (decreased SaO_2_, Hb, or CO) must be considered and managed. Conversely, before ascribing a decrease in SvO_2_ to decreased TO_2_ and increasing it, causes of increased VO_2_ (pain, stress, and fever) should be considered and managed. Of note, ScvO_2_ is more easily obtainable from a central line placed the in superior vena cava (rather than a right cardiac catheter for SvO_2_) and correlates well with SvO_2_ [61]. Thus, decreased ScvO_2_ in shock, once increased VO_2_ has been managed, reflects increased ERO_2_ compensating for decreased TO_2_, which must be explored. These are the principles underlying EGDT [32].

Increased ScvO_2_ may reflect two situations: either an increase in TO_2_ relative to VO_2_, in a successfully optimized, stabilized, or recovering patient, or a decrease in VO_2_ relative to TO_2_, due to mitochondrial dysfunction [62].

These issues highlight that (1) decreased ScvO_2_ is a marker of inadequate global oxygenation which can only be interpreted by taking into account factors related to VO_2_ increase on one hand and TO_2_ decrease on the other and (2) “normal” ScvO_2_ is not a reliable marker of adequate oxygen transport-to-demand when oxygen uptake may be impaired.

#### 2.4.3. ScvO_2_ and Perfusion

**Figure 1:**
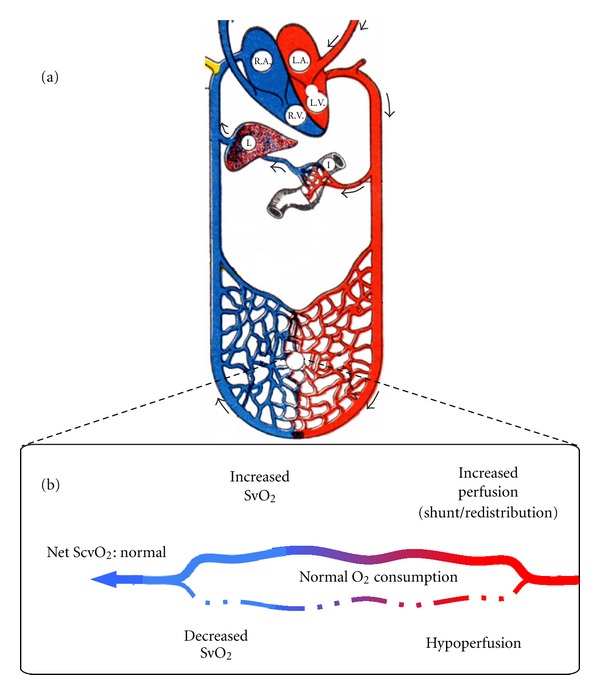
Capillary SvO_2_ and perfusion. (a) Schematic representation of the circulation (arterial in red, venous in blue, R.A: right atrium, L.A: left atrium, R.V: right ventricle, L.V: left ventricle, I: intestine, L: liver) and a generic capillary bed. (b) Schematic representation of both a hypoperfused capillary (lower dashed line) and normally perfused capillary (upper continuous line) receiving increased perfusion redistributed from the hypoperfused capillary. Following normal oxygen consumption by the tissues adjacent to the capillaries, SvO_2_ in each capillary is specified as is the resulting SvO_2_ downstream of the heterogeneously perfused capillaries.

Oxygenation cannot be dissociated from perfusion. Indeed, when global perfusion is decreased due to decreased CO, all circulations have low flow and decreased TO_2_ relative to VO_2_ resulting in decreased ScvO_2_. However, while ScvO_2_-guided therapy reduced mortality in septic shock, 30% mortality remained, due to multiorgan failure with hypoperfusion [32]. The most likely reason for this discrepancy is the inability of ScvO_2_ to explore locoregional or microcirculatory perfusion. Indeed, perfusion heterogeneity, such as in septic shock [63], will lead to hypoxia in tissue surrounding nonperfused capillaries [64]. However, capillaries remaining perfused will receive additional shunted flow from nonperfused capillaries, and, since surrounding oxygen consumption is unchanged, resulting net venous capillary oxygen saturation will be a mix of highly saturated from open capillaries and low saturations from closed capillaries (Figure 1), with a normal net ScvO_2_. 

This also occurs locally with some circulations hypoperfused while contributing little desaturated blood to venous return, and others maintained through macrocirculatory optimization contributing much overly saturated venous blood, again resulting in a net normal ScvO_2_ despite overt or occult hypoperfusion.

Therefore, ScvO_2_ cannot *see* local/microcirculatory hypoperfusion, and normal ScvO_2_ should not be considered the ultimate endpoint [65].

### 2.5. Lactate Clearance

Glycolysis produces pyruvate, which either enters aerobic mitochondrial respiration requiring oxygen or, in tissue hypoxia, is transformed into lactate metabolized by the liver, kidneys, and skeletal muscle. In low flow, increased lactate is related to tissue hypoxia by hypoperfusion [66, 67]. In sepsis, increased glycolysis and increased production by the gut, lung or even white blood cells are thought to participate in nonhypoxic lactate increase [68]. Regardless of metabolism [68] and catecholamine effects on lactate metabolism [69], lactate clearance seems a useful endpoint.

De Backer et al. studying local sublingual capillary perfusion in patients with septic shock showed that lactate clearance was correlated to capillary reperfusion following dobutamine independently of cardiac index, arterial pressure, systemic vascular resistance, or VO_2_ [70]. Lactate clearance may therefore reflect occult hypoperfusion. Indeed, persistent hyperlactatemia has been considered to reflect occult hypoperfusion in studies showing associated with poor prognosis and hypoperfusion-related complications in trauma [71, 72], cardiac arrest [73, 74], septic shock [75, 76], and high-risk surgery [77]. Therefore, lactate clearance has repeatedly been proposed as a resuscitation endpoint, additional or alternative to ScvO_2_.

Simultaneous ScvO2 and lactate clearance were also measured in a study of 203 patients with septic shock in which reaching only the ScvO_2_ goal was inferior to reaching only the lactate clearance goal [78]. This suggests that ScvO_2_ and lactate clearance must be used hierarchically. Interestingly, Rivers participated in a noncomparative study prior to EGDT in which both ScvO_2_ and lactate clearance were used as subsequent endpoints and allowed a low mortality rate of 14% [79].

In the largest RCT comparing two EGDTs in septic shock, Jones et al. showed that ScvO_2_ or lactate clearance performed similarly and concluded that lactate clearance could be used instead of ScvO_2_ [80].

However the real question is not whether lactate clearance should replace ScvO_2_, but if it should be an additional endpoint. Strikingly, while Jones et al. did not find any difference when replacing ScvO_2_ by lactate clearance, Nguyen et al., in a study of sepsis bundles, showed that by adding lactate clearance to ScvO_2_, mortality decreased even further from 24.5% to 17.9% [75].

### 2.6. Venous-to-Arterial CO_2_ Gradient

#### 2.6.1. CO_2_ Production and Transport Physiology

CO_2_ is a byproduct of oxidative metabolism. Tissue production of CO_2_ (VCO_2_) is related to oxygen uptake:

(5) $$\begin{array}{c} {\text{VCO}}_{2}=R\times {\text{VO}}_{2}, \end{array}$$

in which *R* is the respiratory quotient which ranges from 0.7, for pure fat, to 1.0, for pure carbohydrate, as is usually the case in patients with shock; therefore,

(6) $$\begin{array}{c} {\text{VCO}}_{2}={\text{VO}}_{2}, \end{array}$$

(7) $$\begin{array}{c} {\text{VCO}}_{2}=\text{CO}\times \left({\text{CaCO}}_{2}-{\text{CvCO}}_{2}\right), \end{array}$$

(a) $$\begin{array}{c} {\text{VCO}}_{2}=\text{CO}\times k\times \text{P(v-a)}{\text{CO}}_{2}, \end{array}$$

in which P(v-a)CO_2_ is the venoarterial PCO_2_ gradient and *k* the coefficient between CO_2_ concentrations and partial pressures.

#### 2.6.2. Determinants of P(v-a)CO_2_

The previously mentioned Equation (a) can be transformed:

(b) $$\begin{array}{c} \text{P}\left(\text{v-a}\right){\text{CO}}_{2}=\frac{{\text{VCO}}_{2}}{\left(\text{CO}\times k\right)}. \end{array}$$

**Figure 2:**
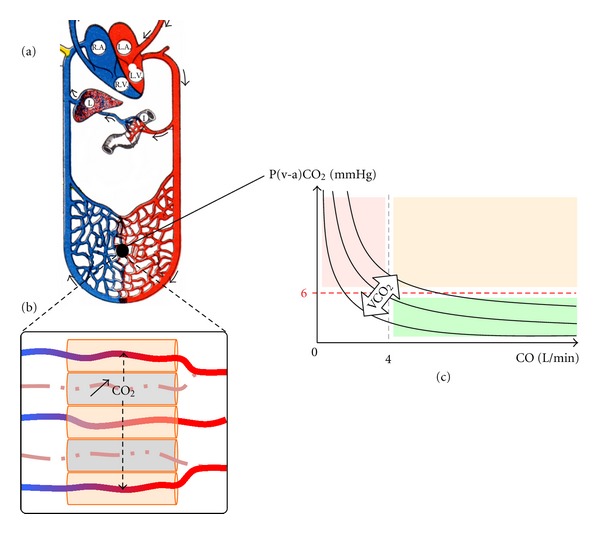
Venoarterial PCO_2_ gradient: relationship to cardiac output and capillary hypoperfusion. (a) Schematic representation of the circulation (arterial in red, venous in blue, R.A: right atrium, L.A: left atrium, R.V: right ventricle, L.V: left ventricle, I: intestine, L: liver) and a generic capillary bed. (b) Schematic representation of both a hypoperfused capillaries (dashed lines) and normally perfused capillaries (continuous lines) receiving increased perfusion redistributed from the hypoperfused capillary. CO_2_ builds up in the tissue adjacent to hypoperfused capillaries (gray cylinders). Due to its highly diffusible nature, accumulated CO_2_ from hypoperfused tissue diffuses to tissue adjacent to perfused capillaries which successfully “washout” this increased amount of CO_2_ leading to higher venous PCO_2_ than normal and therefore a venoarterial PCO_2_ gradient, P(v-a)CO_2_, higher than the upper norm of 6 mmHg. (c) Relationship between P(v-a)CO_2_ and cardiac output (CO). P(v-a)CO_2_ decreases along an isopleth for a given metabolic production of CO_2_ (VCO_2_). For “normal” cardiac outputs over 4 L/min and normal VCO_2_ (green area), P(v-a)CO_2_ remains under the upper threshold of 6 mmHg. Decreased cardiac output below 4 L/min leads to increased P(v-a)CO_2_ due to insufficient “washout” regardless of capillary perfusion. P(v-a)CO_2_ increases over 6 mmHg in conditions of adequate cardiac output, and normal VCO_2_ is pathological and reflects capillary hypoperfusion (off-isopleth orange area).

Therefore, the venoarterial PCO_2_ gradient is proportional to VCO_2_, itself inversely proportional to the CO_2_ clearance from tissues (washout). Given its diffusible nature CO_2_ washout depends mainly on cardiac output (CO) and tissue perfusion. The determinants of P(v-a)CO_2_ are therefore VCO_2_, CO, and tissue perfusion (Figure 2(c)).

CO_2_ washout is so dependent upon flow that any situation of local or regional low flow due to decreased local perfusion (Figure 2(a)) eventually compounded by decreased cardiac output will (1) increase tissue stagnation of CO_2_ (Figure 2(b)) and (2) increase diffusion of CO_2_ from hypoperfused tissue to venous capillaries with residual minimal flow (Figure 2(b)), leading to an increase in P(v-a)CO_2_ >6 mmHg (Figure 2(c)). 

Teboul et al. demonstrated the role of cardiac output in CO_2_ clearance in patients with chronic heart failure and low cardiac output in whom P(v-a)CO_2_ >6 mmHg decreased to normal following dobutamine [81]. Vallet et al. demonstrated, in isolated-perfused canine hindlegs, that P(v-a)CO_2_ increased in conditions of perfusion dependency [82]. This has also been shown through tissue-to-arterial PCO_2_ differences correlated to hypoperfusion [83].

The relationship between P(v-a)CO_2_ and cardiac output is curvilinear, with asymptotic VCO_2_ isopleths (Figure 2(c)): increases in P(v-a)CO_2_ occur when cardiac output decreases, and P(v-a)CO_2_ remains normal when cardiac output is normal or increased. These are major issues for interpretation: (1) decreased cardiac output will increase P(v-a)CO_2_ >6 mmHg independently of underlying hypoperfusion (pink area, Figure 2(c)); (2) increase in P(v-a)CO_2_ >6 mmHg may unmask occult hypoperfusion only if cardiac output is normal or increased (orange area, Figure 2(c)). This second situation arises in resuscitated septic shock in which fluid loading and vasopressors have increased cardiac output without treating underlying septic hypoperfusion [84].

#### 2.6.3. P(v-a)CO_2_ Increase and Clinical Hypoperfusion

Mekontso-Dessap et al. studied 89 critically ill patients with normal cardiac index (IC = 3,65 ± 1,65 L/min/m^2^) seeking to discriminate patients with or without hypoperfusion defined as blood lactate >2 mmol/L. Neither SvO_2_ nor mixed venous PvCO_2_ was discriminant. However, increased P(v-a)CO_2_ was correlated to increased blood lactate levels with an optimal cutoff at 6 mmHg [85].

This was also shown, by Creteur et al., in patients with resuscitated septic shock and normal cardiac index (IC = 3,6 ± 0,6 L/min/m^2^) using in vivo sublingual microcirculation imaging and sublingual tonometric assessment of PCO_2_, in which sublingual PCO_2_-PaCO_2_ difference was correlated to hypoperfusion and decreased with reperfusion following low-dose dobutamine [37]. Vallee et al. studied 56 patients with EGDT-resuscitated septic shock further resuscitated to decrease hyperlactatemia while maintaining ScvO_2_ >70% [86]. Despite normal cardiac index, patients with increased P(v-a)CO_2_ >6 mmHg had slower and lower lactate clearance and increasing organ failure than patients with normal P(v-a)CO_2_.

This prognostic value of P(v-a)CO_2_ was tested in high-risk surgery EGDT showing that ScvO_2_ and P(v-a)CO_2_ were correlated to postoperative complications [87]. Interestingly, complications undetected by “normal” ScvO_2_ (>70%) were detected by increased P(v-a)CO_2_. 

It appears that increased P(cv-a)CO_2_ in resuscitated septic shock or high-risk surgical states may (1) reflect inadequate cardiac output, and, (2) in patients with normal/increased cardiac output, increased P(cv-a)CO_2_ may reflect underlying occult hypoperfusion; (3) targeting P(cv-a)CO_2_ <6 mmHg might be of benefit although it remains unclear how best to achieve this [84].

The future of perfusion monitoring may be comprehensive EGDT-like approaches integrating endpoints of global oxygenation such as ScvO_2_, adequacy of cardiac output to perfusion such as P(cv-a)CO_2_, global perfusion such as lactate clearance and local perfusion indices. All the clinical tools already exist; however, while some can be monitored continuously such as ScvO_2_, others such as P(cv-a)CO_2_ and blood lactate require repeated sampling and blood gas analysis. What remains in order to encourage development of tools for continuous perfusion monitoring through these parameters is to design and carry out studies implementing comprehensive, stepwise, multiple-endpoint, EGDT-like approaches.

## 3. Temperature Monitoring

Maintenance of normal body temperature is critical in the intensive care setting and should be regularly monitored. While assessment of core temperature is ideal, there are other sites that can be used in critically ill patients, and understanding the limitations of any device and the site monitored is essential for clinical decision making.

Indeed, numerous trials have shown that even mild hypothermia causes numerous adverse outcomes [88] including morbid myocardial outcomes [89] secondary to sympathetic nervous system activation [90], surgical wound infection [91, 92], coagulopathy [93, 94], delayed wound healing [91], delayed post-anesthetic recovery, prolonged hospitalization [47], shivering [95], and patient discomfort [96]. In this sense, it is also known that all general anesthetics produce a profound dose-dependent reduction in the core temperature secondary to impairment of normal thermoregulatory mechanisms, the largest culprit being core-to-peripheral redistribution of body heat. 

Core temperature relates to the compartment that is composed of highly perfused tissues whose temperature is uniform. This fact makes that accurate measurement of this temperature has been shown in the pulmonary artery, distal esophagus, tympanic membrane, or nasopharynx [97, 98]. However each of these modalities has their limitations. Esophageal monitoring requires correct positioning at or below the position of maximal heart sounds if esophageal stethoscope is used. Nasopharynx (correctly placed a few cm past the nares) requires obstruction of airflow to prevent the air currents from cooling the thermocouple. Correct tympanic membrane monitoring may be difficult secondary to tortuous aural canal and also requires obstruction of airflow [88]. Finally pulmonary artery catheterization is a highly invasive procedure.

Since these sites are always available or convenient, a variety of “near-core” sites are also used clinically. These include the mouth, axilla, bladder, rectum, and skin surface, all of which have their own limitations. Oral temperature can be inaccurate secondary to recent PO intake and airflow. Axillary temperature may be accurate with correct positioning (over the axillary artery with the patients arm kept by their side) [99]. However difficulty with maintaining this position has limited its use [100]. Rectal temperature has shown to lag behind the core temperature sites and has shown to fail to increase appropriately during certain hyperthermic crises [88, 101–103]. Bladder temperature is strongly affected by urine flow, and it has shown to be equal to rectal temperature when urine flow is low, but equal to pulmonary artery temperature (and thus core) when flow is high [104]. Finally, skin temperature is considerably lower than core temperature [105]. For instance, forehead skin temperature is typically 2°C cooler than core [62], and this gradient may be increased in case of hypoperfusion.

## 4. Respiratory Monitoring of the Ventilated ICU Patients

### 4.1. Introduction

Monitoring of the respiratory system is integral to the daily ICU care of all ventilated patients. Such monitoring in its broader sense includes serial assessment of gas exchange, of respiratory system mechanics, and of patients' readiness for liberation from invasive positive pressure ventilation. Tracking respiratory system changes over time may help minimize ventilator-associated complications, optimize patient-ventilator synchrony, and provide important clues regarding possible causes for alarm sounding and/or changes in patients' conditions. A prerequisite for such an approach is a good understanding of the physiology behind the variables being monitored. 

Despite the importance of respiratory monitoring in ventilated ICU patients, this is not always performed as often or interpreted accurately particularly by some residents and younger colleagues. This is probably due to the general prejudice that some measurements are cumbersome to obtain and/or to interpret in part due to increased role of protocols and to the decreased understanding of physiology specifically at the bedside. Other measurements are taken for granted (e.g., pulse oximetry), and the limitations of the methods are not always taken into consideration. In this paper, our goal is to give a brief overview of key basic readily available parameters and the principles underlying their alterations. These parameters related to respiratory mechanics and gas exchange should be obtained at initiation of mechanical ventilation and at regular interval thereafter particularly in patients who are difficult to ventilate and oxygenate and require heavy sedation and paralysis. These patients have a higher risk of complications, and adequate monitoring becomes even more critical. 

### 4.2. Basic Respiratory System Mechanics

While certain measures require an active patient (e.g., measure of respiratory muscle strength), most bedside measures and estimations of the respiratory system (RS) mechanics require a passive patient. Modern ventilators display real time pressure, volume, and flow time curves. Reviewing these curves daily is essential to assess whether the ventilator settings are safe and adapted to the patients' conditions. 

Partitioning the contribution of the lung from that of the chest wall to the RS mechanics would require measuring pleural pressures and placement of an esophageal probe. Although this measure is not done routinely, understanding and considering chest wall contribution to mechanics are still required. Let us now review key selected measures that should be routinely obtained at the time of initiation of ventilation and thereafter in the passively ventilated patient.

The relationship between pressure, flow, volume, and the mechanics of the respiratory system is best approached using the simplified equation of motion [106] which states that the pressure (*P*) needed to deliver a tidal volume can be calculated as follows:

(8) $$\begin{array}{c} P=\left(\frac{V_{T}}{C_{\text{RS}}}\right)+\left(\frac{R_{\text{RS}}\times V_{T}}{\text{Ti}}\right)+\text{total PEEP}, \end{array}$$

where *V*_*T*_ = tidal volume, *C*_RS_ and *R*_RS_ = overall compliance and resistance of the respiratory system (RS), respectively, Ti = inspiratory time and *V*_*T*_/Ti = inspiratory flow and PEEP = positive end expiratory pressure. 

In a passively ventilated patient, the pressure measured at the airway opening (*P*_ao_) is equal to pressure generated by the ventilator (*P*_*v*_). If the respiratory muscles are actively contributing to inspiration, then *P*_ao_ = *P*_*v*_ – *P*_mus_ (negative intrathoracic pressures generated by the inspiratory muscles). The equation of motion remains valid when mechanical ventilation is delivered by using primarily a volume or a pressure-controlled mode. In the former mode, volume is the set (independent) variable, and pressure becomes the dependent variable whereas in the latter mode pressure is the set and volume is the dependent variable. The equation of motion clearly stresses that the pressure needed to deliver a given *V*_*T*_ is the sum of three distinct pressures that have to be offset: (1) elastic pressure (*V*_*T*_/*C*_RS_), (2) resistive pressure (*R*_RS_ × *V*_*T*_/Ti), and (3) the pressure already present in system at the end of expiration (total PEEP = auto PEEP + external PEEP).

### 4.3. Static Compliance of the Respiratory System

*C*_RS_ is determined by the compliance of both the lung (*C*_*L*_) and the chest wall (*C*_*w*_). It is measured by applying an inspiratory pause long enough (1.5–2 sec) to allow the *P*_ao_ to reach zero flow condition to ensure that *P*_ao_ = plateau pressure (*P*_plat_) = alveolar pressure (*P*_alv_). When flow = *V*_*T*_/Ti = 0, then rearranging the equation of motion allows to calculate *C*_RS_ = *δV*_RS_/*δP*_RS_ = *V*_*T*_/(*P*_plat_ − total PEEP). Note that *C*_RS_ bears a complex relationship to the lung and chest wall compliance since chest wall and lung are in parallel: 1/*C*_RS_ = 1/*C*_*L*_ + 1/*C*_*w*_ and *C*_RS_ = (*C*_*L*_ × *C*_*w*_)/(*C*_*L*_ + *C*_*w*_) [107]. 

To calculate *C*_RS_, *V*_*T*_ should ideally be corrected for the compressed gas in the circuit. This correction is rarely done clinically and probably not needed to simply track *C*_RS_ unless one operates at very high airway pressures, the circuit tubing is quite distensible, or one changes the type of tubing between measures. It is important, however, to use total PEEP and not simply the external PEEP for this calculation and to keep in mind that the distending pressures for the lung are in reality the transpulmonary pressures (*P*_tp_ = *P*_plat_ − *P*_pl_) not simply *P*_plat_. The importance of thinking in terms of transpulmonary pressure lies in the fact that the latter is instrumental in causing lung overdistension and injury when excessive. Since pleural pressure is not routinely measured, interpreting airway pressure requires to consider the contribution of the chest wall and inspiratory muscle to the pleural pressure to estimate the transpulmonary pressure associated with a given airway pressure. For instance, a 35 cm H_2_O *P*_plat_ in patients with morbid obesity or high intra-abdominal pressure (low *C*_*w*_) that elevates *P*_pl_ (e.g. 10 cm H_2_O) is associated with a lower *P*_tp_ (25 cm H_2_O) than the same *P*_plat_ in a patient with normal chest wall compliance actively inspiring with a *P*_pl_ of −5 cm H_2_O (*P*_tp_ = 40 cm H_2_O).

It is important not to equate a change in static *C*_RS_ with an alteration in the intrinsic elasticity of the lung. As demonstrated by Gattinoni et al. [108], the elastic property (1/*C*_*L*_) of the aerated lung in patients with ARDS remains normal (normal specific compliance *C*_*L*_/FRC). The overall low measured *C*_RS_ is thus mainly the result of a reduction in the effective lung volume in this population. In other words, *C*_RS_ tracks the volume of aerated lung available for ventilation or the size of the “baby” lung. The drop in the static *C*_RS_ observed following the accidental migration of the endotracheal tube in the right main bronchus best illustrates this. In addition, since *C*_RS_ is the slope of the pressure-volume curve of the RS which tends to become nonlinear and to flatten at low and high lung volume (upper and lower inflection points describing larger pressure change for a given volume change), *C*_RS_ tends to be the highest around FRC and to decrease at high lung volume if the system becomes overdistended or at low lung volume with the loss of aerated unit (derecruitment). Changes in *C*_RS_ may thus reflect change in the position of tidal ventilation relative to inflection points on the pressure volume curve and/or shift of curve. In conclusion, *C*_RS_ therefore is helpful to size the tidal volume relative to the size of the baby lung and to track if recruitment, derecruitment, or overdistension may occur over time. The stress index proposed to monitor ARDS patients ventilated with constant flow using the shape of the pressure time curve applies the same principle to detect tidal recruitment and overdistension [109].

 For a practical standpoint, measuring *C*_RS_ can provide useful information to set tidal volume relative to the size of the lung. Tracking its change over time is helpful to alert the possibility that derecruitment, overdistension (decreasing *C*_RS_), or recruitment (increased *C*_RS_) is taking place. Everything else being equal, this can be done as often as needed by monitoring *P*_plat_ as long as the patient remains passive and the ventilator settings are the same. *P*_plat_ is an important variable that reflects alveolar pressure and is often used at the bedside to estimate the risk of ventilator-associated lung injury. *P*_plat_ has been found to be associated with outcome in ARDS [110, 111]. Any significant change in *P*_plat_ therefore warrants a thorough assessment of the patients using the principles outlined previously, and one has to incorporate in this process consideration for the pleural pressure.

### 4.4. Resistance of the Respiratory System

Flow (*Q*) and Pressure Drop (ÄP) across the airways are used to calculate the resistance *R*_RS_ = *Q*/ÄP. Since flow occurs during inspiration and expiration, resistance can be defined as *R*_RSI_ and *R*_RSE_. 

By applying an inspiratory pause as indicated previously, airway pressure drops from its peak value (*P*_peak_) to *P*_plat_, and *P*_peak_ − *P*_plat_ tracks the resistive pressure that must be overcome to deliver *V*_*T*_ at a given flow. If flow during inspiration is known, *R*_RSI_ can be easily calculated. More pragmatically, it is important at initiation of ventilation to measure *P*_peak_ and *P*_plat_ and to make note of the pressure difference between those two pressures to assess whether the patient may have abnormal airway resistance (large *P*_peak_ to *P*_plat_ difference), keeping in mind that an inappropriately high set flow rate or a small endotracheal tube may both increase this pressure difference. The initially recorded *P*_peak_ and *P*_plat_ difference will then allow one to monitor for any change in *C*_RS_ and *R*_RSI_ and to establish when facing a *P*_peak_ pressure alarm (during volume controlled ventilation), if a *P*_peak_ change is due to a compliance or resistance alteration. For instance, a sudden increase in peak pressures associated with a larger *P*_peak_ to *P*_plat_ difference is most consistent with an increase in resistance secondary to the native airway problem (e.g., bronchospasm) or partial obstruction of the artificial airways (e.g., ET tube kinked or obstructed by secretions.) In contrast, an unchanged *P*_peak_ to *P*_plat_ difference strongly supports a change in static *C*_RS_ (e.g., tension pneumothorax, right main bronchus intubation, atelectasis, or pulmonary edema) as the cause of the *P*_peak_ pressure alarm.

Expiratory flow and airway resistance vary with lung volume and flow decays exponentially in normal circumstances. The endotracheal tube, exhalation valve, heat moisture exchangers—when present—as well as the native airways all contribute to the expiratory resistance (*R*_RSE_). A first important step is thus to identify the site responsible for any abnormal airway resistance. *R*_RSE_ is a parameter that is neither easy nor necessary to measure routinely. What is always needed, however, is to recognize the presence of an abnormally high resistance, to identify and treat its cause, and to monitor and minimize its consequences. Consequence could be dynamic hyperinflation and auto-PEEP, which increases the work of breathing and the risk of barotraumas and/or hypotension [112]. Abnormally high expiratory flow resistance can easily be recognized by observing that the shape of flow time curve becomes biexponential (flow limitation) and that the expiratory phase is prolonged, and flow does not reach zero before the next tidal breath is delivered by the ventilator or initiated by the patient. The leads to dynamic hyperinflation auto-Peep and commonly wasted inspiratory effort and asynchrony. 

### 4.5. Dynamic Hyperinflation

As discussed previously, dynamic hyperinflation is important to monitor and recognize. Measuring auto-PEEP and *P*_plat_ at the initiation of the ventilation and at regular intervals helps with the detection of dynamic hyperinflation. The pressure measured at the end of expiration when airflow is interrupted is termed total PEEP. Auto-PEEP is then calculated as the difference between total PEEP and extrinsic PEEP (PEEP set on the ventilator). Most modern ventilators have the capacity to measure auto-PEEP semiautomatically. Auto-PEEP may develop for a variety of reasons (e.g., airflow obstruction, high lung compliance, high minute ventilation, and whenever the ventilatory settings are such that expiratory time is insufficient for lung volume to return to its relaxed FRC). 

Auto-PEEP does not necessarily mean that dynamic hyperinflation is present. It is thus not synonymous with dynamic hyperinflation. If a patient is actively expiring, the calculated auto-PEEP may merely reflect active expiration and not necessarily the degree of hyperinflation, if any. This can be detected by observing the patient and by placing a hand on the patient's abdomen to feel for contraction of the abdominal muscle during expiration and the measurement. 

In addition, even in a passive patient, auto-PEEP may underestimate the degree of hyperinflation. Auto-PEEP is a measure of the average positive pressure present in the system at the end of expiration. Some lung regions with high auto-PEEP may not contribute to the average auto-PEEP measured due to airway closure (hidden PEEP) [113]. This prevents accurate assessment of alveolar pressure at the end expiration in all lung regions. When hidden PEEP is present, the overall degree of hyperinflation present will be reflected during tidal ventilation and thus in the *P*_plat_, and the tidal volume is delivered on top of the trapped gas. It is therefore important to monitor both auto-PEEP and *P*_plat_ in patients with obstructive physiology and to adjust the ventilator to minimize dynamic hyperinflation and address its cause. This often requires decreasing minute ventilation and accepting some degree of respiratory acidosis (permissive hypercapnia). Sometimes increasing the external PEEP helps reduce airway collapse during expiration and reduce the work needed to trigger the ventilation. When PEEP is used in this setting, it is typically set at a level below the measured total PEEP but one subsequently measures the resulting changes in *P*_plat_ and trapped volume, as the effects of external PEEP on *P*_plat_ are difficult to predict [114]. 

### 4.6. Gas Exchange 

#### 4.6.1. Monitoring Oxygenation

The adequacy of tissue oxygen delivery and utilization cannot be measured directly, and the oxygenation status of vital organ is typically inferred and monitored by using data from different sources. 

*Arterial Oximetry.* Oximetry is a widely used monitoring technique in ICU. Despite its accepted utility, it is not a substitute for arterial blood gas monitoring as it provides no information about the ventilatory status and has several other limitations. Probe placement is important as both high and low values could be seen with partial alignment of the probe electrodes [115], presence of the blood pressure cuff on the same side as the oximetry probe [116], excessive motion (e.g., shivering or seizures) [117, 118], and having electromagnetic fields such as those created by MRI machines, cellular phones, and electrocautery [119, 120]. 

Erroneous readings may also be caused by hypotension [121] and hypoperfusion due to hemodynamic instability and use of vasoconstrictor medications [122, 123]. Forehead sensors may be more accurate in those circumstances. Abnormal hemoglobin moieties such as methemoglobin [117, 124, 125] and carboxyhemoglobin [115, 117, 126, 127] could result in overestimation of oxyhemoglobin. False readings are also seen in severe anemia (Hb < 5 g/dL) [128], in presence of excessive skin pigmentation [115, 129], nail polish [119], or dyes. It is thus important to question the reading when the latter does not seem to fit the clinical picture. 

*Efficacy of Oxygen Exchange. *Oximetry is not a sensitive guide to gas exchange in patients with high baseline PaO_2_ because of the shape of the oxygen dissociation curve. On the upper horizontal portion of the curve large changes in PaO_2_ may occur with little change in pulse oximetry (SpO_2_) [130] till the PaO_2_ is in the mid sixty range. It is thus wise to adjust the inspired O_2_ to keep the hemoglobin saturation below 100 percent. Numbers of indices have been used to assess the efficiency of oxygen exchange including venous admixture and shunt fraction. The calculation of these indices involves mixed venous blood sampling with a PA catheter, which are not commonly used anymore in most centers. These indices are thus more helpful for research than for daily care. Alveolar-arterial oxygen tension difference has been used in the past but it is limited, and it changes unpredictably with FiO_2_ changes in critically ill patients with combination of etiologies of hypoxia. 

Conditions encountered in the ICU associated with a low PaO_2_ include (1) hypoventilation, (2) impaired diffusion, (3) ventilation/perfusion mismatching, and (4) shunting. Significant shunting as opposed to ventilation/perfusion mismatching is likely present if 60% or greater FiO_2_ is required to keep the arterial O_2_ saturation above 90%. Since PaO_2_ is loosely related to gas exchange efficiency unless the FiO_2_ is also taken into consideration, the PaO_2_ : FiO_2_ ratio is thus generally used to quantify the degree of pulmonary gas exchange dysfunction and lung injury. Indeed, this ratio is integral to the definition of ALI and ARDS [131]. PaO_2_ : FiO_2_ ratio in the 500–300 range is consistent with normal-to-mild impaired oxygen exchange; values less than 300 indicates moderately impaired gas exchange as seen in ALI, and values of less than 200 are supportive of significant shunt physiology as encountered in ARDS. The ratio can also be used to assess the response to therapeutic interventions [132, 133].

Although in one study [134] PaO_2_ : FIO_2_ ratio exhibited stability at FiO_2_ values of ≥0.5 and PaO_2_ values of ≤100 torr (≤13.3 kPa), others have found the PaO_2_ : FiO_2_ ratio to have poor association with pulmonary shunt [135] and that alteration in the PaO_2_ : FiO_2_ occurred when the FiO_2_ is changed [136]. Such variability makes this parameter dependent on the management style: for example, aiming to keep the arterial O_2_ saturation on the high side (e.g close to 99%) as opposed to the low side (e.g., close to 90%) may cause certain patients to have to be reclassified from ARDS to ALI. Another important limitation of the PaO2 : FiO2 ratio to assess the gas exchange function is that it is also affected by the ventilatory strategy such as the size of the tidal volume used [110], the PEEP level and presence of recruitable lung regions [137], and the hemodynamic conditions. PaO_2_ : FiO_2_ ratio has not been shown to correlate with the mortality in ARDS/ALI [138]. Despite its limitation, when used in combination with other hemodynamic and respiratory mechanics measures, monitoring PaO_2_ : FiO_2_ ratio is easy to obtain at the bedside to track cardiorespiratory changes. It is important to keep in mind the above limitations, its lack of correlation with outcome, and that the overall goal of mechanical ventilation is to achieve acceptable gas exchange with minimal stress and not to achieve the highest PaO_2_ : FiO_2_ ratio as such strategy has the potential to be counterproductive. 

#### 4.6.2. Monitoring Carbon Dioxide

Arterial PCO_2_ depends on CO_2_ production relative to its elimination by the lungs. In sedated and passively ventilated patients who have a fixed imposed minute ventilation, PCO_2_ may rise due to increased metabolic rates such as seen, for instance, with fever, a high carbohydrate load, and/or overfeeding. Blood PCO_2_ level is also governed by acid-base fluctuations and perfusion adequacy. Finally, PCO_2_ may rise if alveolar ventilation decreases as dead space increases. We shall now briefly discuss monitoring of PCO_2_ and dead space. 

*Assessment of *PaCO_2_. Traditionally in the ICU, gas exchange is assessed on the arterial side by measurement of PaO_2_, PaCO_2_, and pH. There has been a long-standing interest in alternate methods for measurement of PaCO_2_. 

PaCO_2_ can be continuously monitored using miniaturized electrochemical or optical sensors. End-tidal CO_2_ (etCO_2_) and transcutaneous PCO_2_ (*tcPCO*_2_) are commonly used in operative rooms and sleep centers. *tcPCO*_2_ measurement uses a sensor to detect CO_2_ that is diffusing out through the body tissues and skin and could be a helpful alternative to blood gas measurement. The *tcPCO*_2_ measured by this technique measures tissue CO_2_ that is slightly higher than the arterial value requiring corrective algorithms. *tcPCO*_2_ can be used to estimate and to trend PaCO_2_ in different settings such as adult critical care [139, 140], mechanically ventilated patients [141, 142], and pediatric and neonatal ICU [141, 143]. However, the accuracy of *tcPCO*_2_ measurement is limited during severe vasoconstriction or presence of skin edema. Other limitations include the need for periodically changing the membrane and calibrating the sensor when using electrochemical measurement technique. 

Recent publications [144, 145] have evaluated the role of measurement of PaCO_2_, PaO_2_, pH in peripheral and central venous blood instead of arterial blood. In the studies venous PCO_2_ and pH were a reasonable surrogate of arterial PCO_2_ and pH. In normal conditions venous PCO_2_ is 3-4 mmHg higher than the arterial blood that leads to an increase in bicarbonate levels (1–1.5 mmol per liter) and a simultaneous decrease in a pH by 0.03–0.05 pH units. However, in the presence of shock or cardiac arrest the arterial-to-venous PCO_2_ and pH difference increases. Such an increase in difference may be an important clue that tissue hypoperfusion is present, and the case has been made that in cardiac arrest patient venous blood gas may better reflect tissue acid-base status and oxygenation than arterial blood gas [146]. 

*Dead Space Ventilation and *PCO_2_ *in ICU. *The physiologic dead space (*V*_*D*_) refers to the portion of tidal breath, which fails to participate in effective CO_2_ exchange and is made of the sum of the “anatomic” and the “alveolar” dead space. The dead space fraction can be estimated by simultaneous measurement of arterial PCO_2_ and partial pressure of exhaled gas CO_2_:

(9) $$\begin{array}{c} \frac{V_{D}}{V_{T}}=\frac{\left({\text{PaCO}}_{2}-{\text{PeCO}}_{2}\right)}{{\text{PaCO}}_{2}}. \end{array}$$

In ventilated patients, the ventilator circuit increases, and a tracheostomy decreases, the anatomic dead space. Modest decreases in the dead space can also be seen with extended breath holding [147, 148] and decelerating inspiratory flow pattern ventilation [149, 150]. Other common ICU conditions associated with an increased *V*_*D*_ include low cardiac output states, pulmonary embolism, pulmonary vasoconstriction, and mechanical ventilation with excessive tidal volume or PEEP particularly when blood volume is low [151]. 

In critically ill patients, it is not exceptional for the *V*_*D*_/*V*_*T*_ to rise to values that exceed 0.65 (normal 0.35) [152, 153]. Dead space accounts for most of the increase in *V*_*E*_ requirements and CO_2_ retention seen in lung injury and hypoxic respiratory failure. Overdistention leading to increased dead space should be suspected when under controlled constant inspiratory flow ventilation, examination of the pressure time curve demonstrates concavity or an upward inflection. It should be considered in the differential when associated with an elevated *P*_plat_. In these situations, reducing the tidal volume or PEEP could help reduce *V*_*D*_/*V*_*T*_. 

In patients with ARDS, increased dead space, rather than a decrease in PaO_2_ : FiO_2_ (oxygenation), has been shown to be associated with alteration of the lung structure [108] and increased mortality [153–156]. It is not known if therapy or ventilatory strategy aiming at reducing dead space would improve ARDS patient's outcome. 

In ARDS, hypercapnia could result from lung protective ventilation (permissive hypercapnia), due to increased dead space due to damaged lung or a combination of both. It is important to differentiate respiratory acidosis due to increased dead space associated with an elevated minute ventilation and mortality from the one that results from a lung protective strategy (permissive hypercapnia) associated with lower mortality [157] and deliberately low tidal volume. Although respiratory acidosis *per se* may have a lung protective effect in experimental ventilator-induced lung injury model [158] and in patients exposed to high mechanical stress [159], respiratory acidosis has complex biological effects and is not without potential hazards, as reviewed elsewhere [160, 161]. In the absence of contraindication, respiratory acidosis is currently justified only to limit injurious mechanical stress or dynamic hyperinflation.

In summary, monitoring of oxygenation and ventilation is important but before attempting to adjust the ventilator to correct the PaO_2_ and/or PaCO_2_ to normal levels, the underlying alteration in the respiratory physiology and mechanics needs to be understood and its cause addressed whenever possible. It is also essential to weigh the risk benefits specifically in regard to mechanical stress on the lungs before attempting to correct abnormal blood gas values. Monitoring in the ICU should aim to keep the patients within a safety zone and does not imply we need to act on all abnormal values. First do no harm.

## 5. The Monitoring of the Nutritional and Metabolic Care in the Intensive Care Unit (ICU)

**Figure 3:**
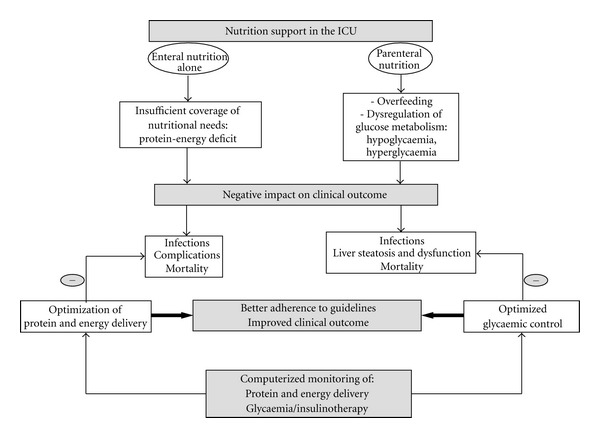
Conceptualization of the expected impact of the computerized monitoring of the nutritional and metabolical care in the intensive care unit (ICU). Although it is the recommended nutrition support, early enteral nutrition (EN) is associated with an insufficient coverage of energy and protein needs, leading to a protein-energy deficit, itself associated with an increased risk of infections and complications and increased mortality. The use of parenteral nutrition (PN) could be associated with overfeeding, and especially hyperglycaemia, which is associated with an increased risk of infections and liver metabolic complications and increased mortality. By allowing an early and tight adaptation of protein and energy delivery to nutritional targets and an optimization of glycaemic control, the computerized monitoring of the nutritional and metabolical care could improve the adherence to clinical guidelines and the clinical outcome of ICU patients.

The monitoring of nutritional and metabolic care in the ICU has three main goals: first, the control of macronutrients (glucose, protein, fat) and micronutrients (vitamins and trace-elements) delivery, second, the assessment of the adequation between energy needs and delivery, and, finally, the glycaemic control. This issue is of high relevance, since a plenty of evidence indicates that an insufficient coverage of protein and energy needs and an impaired glycaemic control are both related to a worse clinical outcome in the ICU. Several studies have demonstrated that computer-assisted systems allow an accurate monitoring of nutrition and metabolic parameters and contribute to optimize protein-energy delivery and glycaemic control. Therefore, a daily computerized monitoring of nutrition support could contribute to improve the adherence to guidelines and the clinical outcome of ICU patients (Figure 3).

### 5.1. Monitoring of Protein and Energy Delivery for the Prevention of Protein-Energy Deficit

#### 5.1.1. Rationale

In the ICU, the first line recommended nutrition support is the early enteral nutrition (EN) [162, 163], since it reduces infectious risk and mortality in comparison with late EN [164] and early parenteral nutrition (PN) [165]. Yet, several observational studies have shown that the use of EN during the first week of the ICU stay is associated with a protein and energy deficit [166, 167], which is, in turn, related to an increased risk of infections [166–169] and complications [167], as well as increased mortality [170]. Delivering too much energy regarding the needs, that is, overfeeding, favors the onset of hyperglycaemia and its related complications [171]. Reaching an adequacy between nutritional needs and delivery is mandatory in all ICU patients to avoid protein-energy deficit, overfeeding and hyperglycaemia, and the onset of their related complications.

#### 5.1.2. How Can Protein and Energy Delivery Be Monitored in Clinical Practice?

Current guidelines recommend the use of indirect calorimetry to measure energy needs [162, 163]. In the situations where indirect calorimetry is not available, which is the case in most ICUs, the use of predictive formula, that is, 20–25 kcal/kg/day at the acute phase, and 25–30 kcal/kg/day at the postacute phase, is advised [162, 163]. Because of the absence of measurement methods, protein needs should be evaluated according to the 1.2–1.5 kcal/kg ideal body weight/day formula. Once the energy target is established, energy and protein delivery has to be monitored to prevent the onset of energy deficit. Several studies have shown that the use of computerized systems for the prescription and the monitoring of nutrition support allows decreasing time for prescription and improving the adequacy between delivery and needs of energy, glucose, protein, and fat [172–176]. Recent clinical studies have demonstrated that the computer-assisted optimization of nutrition delivery could improve the clinical outcome of ICU patients [177, 178]. Singer et al. have shown that the computer-assisted targeting of energy delivery according to indirect calorimetry could reduce mortality in comparison with targeting energy delivery according to the 25 kcal/kg/day formula [177]. Also, a study published in an abstract form has suggested that the computer-assisted full coverage of energy target by supplemental PN from the fourth day of ICU stay could reduce the number of infections and the duration of mechanical ventilation in ICU patients covering only 60% of their energy target by EN alone within the three first days of stay [178]. In addition, computerized monitoring systems allow registering gastric residual volumes and could be helpful for the prescription of prokinetics and antioxidant micronutrients. Nevertheless, computerized monitoring alone is not sufficient for an optimal coverage of nutritional needs. It represents a clinical tool helping at implementing the nutritional recommendations in the context of a global educational and interdisciplinary program of nutritional care [175]. One study has shown that, in addition to a computer-assisted global nutritional program, the presence of an ICU-dedicated dietician further improves protein-energy delivery in the ICU [175].

### 5.2. Monitoring of Glycaemia and Insulinotherapy for Optimized Glycaemic Control

#### 5.2.1. Rationale

In the past 20 years, it was extensively demonstrated that PN could induce metabolic disorders, such as hyperglycaemia, hypertriglyceridemia, liver steatosis, endocrine dysfunction, impairment of immunity, infections, and increased mortality [171]. PN-related infectious complications have been related to hyperglycaemia [171]. Large randomized, controlled, prospective studies have shown that an optimized glycaemic control with the aim to obtain a glycaemia less than 10 mmol/L and avoide hypoglycaemia reduces mortality [179, 180]. Therefore, it is now established that, through a daily monitoring of glycaemia and insulin doses, optimized glycaemic control allows improving the clinical outcome of ICU patients.

#### 5.2.2. How Can Glycaemia and Insulinotherapy Be Monitored in Clinical Practice?

Computerized systems have to be used for the constitution of insulin algorithms that have been shown to improve the glycaemic control in comparison with manual protocols [172]. In addition, computerized systems allow reducing nurses and physicians work time, time to reach the targeted glycaemia and the onset of hypo- and hyperglycaemia [172, 181]. For example, a pilot study suggests that nurse-centered computer-assisted glycemia regulation during stepwise increases of PN according to a predefined protocol resulted in adequate caloric intake within 24 hours together with an adequate glycaemic control [182]. Recent articles develop physiological and practical mathematical models for intensive insulin therapy and tight glycaemic control [183, 184]. Moreover, new devices continuously measuring glycaemia using intravascular catheters have been produced recently. This kind of advanced metabolic monitoring technology could be of great help in the future. Further research is needed to identify the most sensitive models for optimal insulinotherapy and glycaemic control.

In summary, the monitoring of the nutritional and metabolical care is part of the management of the ICU patient. The use of a computer-based monitoring system of nutrients delivery and glycaemic control contributes to reinforce the adherence of clinical practice to guidelines. In addition, computer-based monitoring systems, by preventing protein-energy deficit and overfeeding and optimizing glycaemic control, should contribute to improve the clinical outcome of ICU patients. The medicoeconomic impact of computer-based monitoring systems remains to be evaluated. 

## Figures and Tables

**Table 1:** ScvO_2_ variations related to causes of TO_2_ and/or VO_2_ variations.

ScvO_2_ < 70%		ScvO_2_ > 75%	
Increased VO_2_	Decreased TO_2_	Decreased VO_2_	Increased TO_2_
(i) Pain	(i) Anemia	(i) Analgesia, sedation, anesthesia	(i) High Hb
(ii) Anxiety	(ii) Hypoxemia	(ii) Anxiolytics	(ii) Supplemental oxygen ventilation/high FiO_2_
(iii) Fever	(iii) Low CO	(iii) Hypothermia	(iii) High CO
(iv) Shivering	(1) Hypovolemia	(iv) Muscle paralysis	(iv) Mitochondrial dysfunction
(v) Polypnea	(a) Relative	(v) Mechanical ventilation	
(vi) Respiratory distress	(b) Absolute		
(vii) Increased work of breathing	(2) Vasoplegia		
	(3) Myocardial depression		

ScvO_2 _: central venous oxygen saturation; VO_2 _: oxygen consumption; TO_2 _: oxygen transport; CO: cardiac output; Hb: hemoglobin; FiO_2_: inspired oxygen fraction.
